# Can cognitive psychological research on reasoning enhance the discussion around moral judgments?

**DOI:** 10.1007/s10339-016-0760-y

**Published:** 2016-03-25

**Authors:** Michal Bialek, Sylvia Terbeck

**Affiliations:** Department of Economic Psychology, Kozminski University, Jagiellonska 59, Warsaw, 03-301, Poland; University of Plymouth, School of Psychology, Drake Circus, Plymouth, PL1 4AA United Kingdom

**Keywords:** Moral judgments, Dual-process theory, Signal Detection Theory, Reasoning, Default interventionist model, Intuitive logic

## Abstract

In this article we will demonstrate how cognitive psychological research on reasoning and decision making could enhance discussions and theories of moral judgments. In the first part, we will present recent dual-process models of moral judgments and describe selected studies which support these approaches. However, we will also present data that contradict the model predictions, suggesting that approaches to moral judgment might be more complex. In the second part, we will show how cognitive psychological research on reasoning might be helpful in understanding moral judgments. Specifically, we will highlight approaches addressing the interaction between intuition and reflection. Our data suggest that a sequential model of engaging in deliberation might have to be revised. Therefore, we will present an approach based on Signal Detection Theory and on intuitive conflict detection. We predict that individuals arrive at the moral decisions by comparing potential action outcomes (e.g., harm caused and utilitarian gain) simultaneously. The response criterion can be influenced by intuitive processes, such as heuristic moral value processing, or considerations of harm caused.

## Dual-process approach to moral judgments

Fyodor Dostoyevsky in his *Brothers Karamazov* describes a discussion, in which Ivan asks his brother Alyosha:

Tell me – I challenge you: let’s assume that you were called upon to build the edifice of human destiny so that men would finally be happy and would find peace and tranquility. If you knew that in order to attain this you would have to torture just one single creature, let’s say the little girl who beat her chest so desperately in the outhouse, and that on her unavenged tears you could build that edifice, would you agree to do it? Tell me and don’t lie.

No, I would not, Alyosha said softly.

Alyosha’s decision can be described as deontological; a moral rule has to be obeyed no matter the consequences. It follows that one cannot harm a single person even if the whole of humanity would benefit from it. The opposite moral position to deontology is utilitarianism, whereby actions that maximize the general happiness are rated as morally acceptable. Researchers in many fields, including cognitive psychology and neuropsychology, try to find mechanisms underlying each moral position, answering the question: What makes some people utilitarian sometimes and what deontological? They also inquire as to whether people are consequentialist in their judgments, or vary along this dimension depending on circumstances. To investigate this, researchers usually employ a set of moral dilemmas, such as the trolley dilemma (Foot 1978). In this dilemma one has to decide whether one would pull a lever and change the track of an out-of-control trolley, which would save five men but at the cost of one person who stands on the other track. The majority of people decide to pull the lever (Lanteri et al. 2008; Shallow et al. 2011). A modification of this dilemma requires one to push a very heavy man off a footbridge to stop the trolley (Quinn 1989; Thomson 1985) and results in much lower number of utilitarian decisions (Greene et al. 2001, 2004; Petrinovich et al. 1993). This example shows that the majority of people solve each moral problem separately rather than having a strictly defined—deontological or utilitarian—moral position they apply to all dilemmas. This finding encouraged researchers to investigate what constitutes specific moral judgments.

Researchers have proposed the theory that the two components on which decisions are based are intuition (Type 1 processing) and reflection (Type 2 processing). Type 1 processing is fast, automatic, and heuristic, while Type 2 processing is slow, rule-based, and typically requires cognitive resources (e.g., working memory capacity). Both types of cognitive processing can work separately or at the same time but not necessarily at the same speed, and when working simultaneously they may cooperate or be in conflict (Evans and Stanovich 2013; Stanovich 2009). Contemporary discussion is also strongly affected by the work of Jonathan Haidt (Greene and Haidt 2002; Haidt 2001, 2007), suggesting a prominent role for intuition in moral judgments. Haidt suggested that the vast majority of moral judgments are processed intuitively, and reflection mostly serves its role as a post hoc justification. It was further proposed that the moral intuition was deontological. In particular, when an individual engages in controlled deliberation, they usually decide counter to their immediate intuitions and might therefore reach a utilitarian decision (Greene et al. 2004; Paxton et al. 2012).

There is evidence that supports the approach of the reflexive basis for utilitarianism and intuitive character of deontology. For example, it has been reported that people endorse more deontological judgments under time pressure (Suter and Hertwig 2011) and under cognitive load (Bialek and De Neys 2016a; Trémolière and Bonnefon 2014). Furthermore, Conway and Gawronski (2013) proposed that deontological and utilitarian decisions are separate processes, and showed that cognitive load selectively decreases utilitarian decisions. Additionally, it was found that utilitarian decisions are associated with activity in dorsolateral prefrontal cortex, which is also suggested to be associated with higher-order cognitive processes. Furthermore, the tendency to engage in reflection increases the likelihood of utilitarian decisions (Bartels 2008; Paxton et al. 2012). In another study Greene et al. (2008) additionally showed that cognitive load increased the time required to make a utilitarian decision without affecting the time to make deontological decisions. Despite seemingly strong evidence supporting dual-process theories of moral judgments, in the following we will present challenges to this theory.

## Critique of dual-process theories of moral judgments

Imagine a different moral dilemma: Would you, as a doctor, kill one patient and then harvest his organs to use them to cure five other dying patients? Even though this should be rated as morally acceptable from a utilitarian perspective, almost no one rates such an action as morally acceptable. In the pilot to one of our studies (Bialek and De Neys 2016b) we presented individuals with a set of four moral dilemmas, including the doctor scenario. Out of almost 200 participants, only one decided that he would do the action. At the same time however, more than 60 % of the tested individuals declared willingness to pull the lever in the trolley dilemma or to redirect killing fumes to a room with only one patient instead of three. What is (cognitively) required to kill a patient, push the fat man down the tracks, or pull the lever, in order to save five people? Indeed, the “net gain” of lives saved is always the same in those dilemmas, and thus, the willingness to act should be the same. Therefore, we doubt that the reported observation can be explained by a utilitarian preference for greater good. This allows us to ask two questions: Is the unwillingness to sacrifice motivated by (1) deontological morality or (2) by repulsive emotions causing an alarm reaction to the prospect of directly harming a single victim? The main difference between the two motives is that despite the same outcome (no action taken), in the first case a decision is consistent with one’s internal deontological moral beliefs, while in the second case one “could” endorse the utilitarian morality, but emotions effectively block the application of these preferences. Hence, some individuals who are declaratively utilitarian can take utilitarian actions only in some dilemmas (i.e., trolley dilemmas), but not in other, more direct dilemmas (i.e., the doctor dilemma).

Several researchers (Bartels and Pizarro 2011; Kahane et al. 2015) have examined the relationship between moral dilemmas and personality traits. They reported a negative correlation between utilitarian disposition and empathy and a positive correlation between utilitarian disposition and psychopathy. The authors suggested that within the dilemmas, harm aversion is more significant than merely applying utilitarian principles. When the harm caused is indirect or a side effect of an action the chances of making utilitarian judgment increase (Christensen and Gomila 2012). It can thus further be suggested that individuals whose emotional response is blocked or weakened are more willing to sacrifice one person to save more. For example, individuals in trait alexithymia are more often accepting of the sacrifice (Patil and Silani 2014b) or accept accidental harm caused by action (Patil and Silani 2014a). Furthermore, research has also shown that individuals under the influence of alcohol, whose emotional response to causing harm is lowered, tend to be more utilitarian (Duke and Bègue 2015).

In the light of Greene’s model (Greene 2014), one should expect that lowered emotional response decreases the Type 1–Type 2 conflict in favor of the latter and thus results in more utilitarian judgments. However, contrary to these findings, our previously reported research showed that pharmacologically blocked affective responses decreased the willingness to sacrifice one person (Terbeck et al. 2013). This opposes the idea that deontological judgments are cued uniquely or predominantly by emotions. Furthermore, even though Paxton et al. (2012) found that cognitive reflection test scores positively correlated with utilitarian decisions, according to Baron et al. (2015) this correlation is obtained only because CRT correlates with actively open-minded thinking. Thus, Baron argues that “utilitarian judgments arise from a commitment to a utilitarian approach, which exists before subjects come into the experiment.” Therefore, it is suggested that the relationship between cognitive reflection and utilitarian moral inclination might be more complex. For example, Körner and Volk (2014) reported that decreased cognitive capacity is associated with an increased likelihood of making deontological judgments. However, this effect was only found for the concrete construal compared to the abstract, in which this relationship was reversed. The above discussion on moral judgments demonstrates the complexity of the topic, suggesting that further research is required to answer the open questions of cognitive mechanisms involved in moral dilemmas. We find that the majority of theories on moral judgments utilize the dual-process framework without explicitly indicating whether Type 1 and Type 2 processes are working sequentially or in parallel; hence it is not explicitly stated whether Type 2 processes start together with Type 1 processes (i.e., running in parallel), or might, for example, be triggered by some features of the Type 1 processes (i.e., sequential). What is mostly agreed, however, is that Type 1 processes are faster than the Type 2 processes (Kahneman 2011). In the next section we will show how cognitive psychology could enhance the discussion, by discussing intuitive and deliberative processing, logical reasoning, and probabilistic thinking. We have presented some of our arguments in a previous more philosophical article (Bialek et al. 2014), but this work extends the position and discusses the findings in a broader context.

## Cognitive psychology of reasoning

Cognitive psychology studies on decision making also combine reflection and intuition. The most prominent example of this type of problem is the belief bias (Evans et al. 1983). Consider the following example,

All famous musicians are creative.

All people who take drugs are creative.

Therefore, all famous musicians take drugs.

This conclusion is logically invalid, but believable. Typically, in reasoning studies, individuals are presented with a set of premises and a conclusion and then are asked to evaluate the validity of the conclusion. Research has shown that the majority of people prefer believable conclusions over valid ones (Newstead et al. 1992). Moreover, individuals usually only reason when the conclusion is unbelievable (i.e., they do not engage in reflection when the conclusion is believable). The conditional willingness to reason is called motivated reasoning (Kunda 1990) and results in increased accuracy when assessing the validity of unbelievable conclusions (Evans et al. 1983; Trippas et al. 2014). The interaction between believability and validity delivers an example for the intuition-deliberation trade-off. Here, researchers focused on conditions under which individuals engage in effortful deliberation to override their intuitive, belief-based response in reasoning tasks. The aim is to understand how people detect that they should engage in deliberation (De Neys and Bonnefon 2013). The similarity of this problem with moral judgments is straightforward, and in the next part of the paper we will show how, after introducing the analogy between believability/morality and validity/utility, the study of reasoning can influence the discussion on moral judgments.

There are two classical explanations of the belief bias: misinterpreted necessity and selective scrutiny (Evans 2007, 2008). Without going into detail about these theories, both focus on the believability of the conclusion as a factor to trigger the deliberative reasoning process. When a conclusion is believable people tend to accept the conclusion without further analysis of its validity; but when the conclusion is unbelievable they tend to search for counterexamples (Johnson-Laird 2012) or analyze the logical structure in order to reject the conclusion (Evans 2007; Klauer et al. 2000). Alternatively, Type 2 processes are triggered by “conflict detection” associated with intuitive response (Pennycook et al. 2015). Similar to this, in moral judgments for example, Haidt (2007) discussed the idea that deliberation is only encouraged if the intuitive response is unsatisfying (analogous to unbelievable), specifically by creating too extreme negative emotional response. However, if the intuitive response is satisfying (analogous to believable) people tend to accept it or look for justifications to increase their own confidence (Haidt 2001). This view on reasoning has been recently challenged by two types of research: Signal Detection Theory and Logical Intuition. We will present these in more details in the next section.

### Signal Detection Theory and response biases

**Fig. 1:**
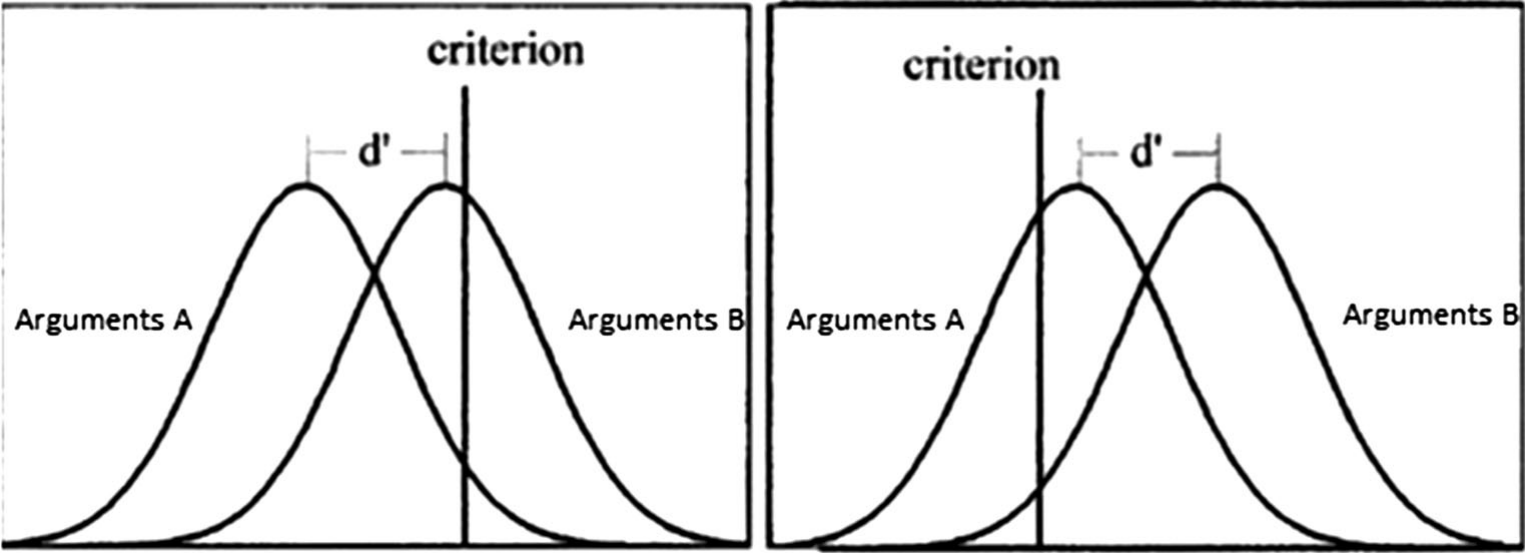
Signal Detection Theory model of reasoning. Criterion is adjusted according to one’s subjective preferences and is affected by believability of a conclusion. The sensitivity index d′ is a measure of similarity of the argument distributions

The interaction between beliefs and logic is important in order for understanding moral judgments, as we need to understand under which conditions deliberation is used to justify (presumably a deontological) intuition and when to reflect on the dilemma in order to override the intuition and draw a (utilitarian) judgment. Sequential models of reasoning underline believability as a factor which is triggering the reflection. Recently Dube et al. (2010) using Signal Detection Theory (SDT) suggested otherwise; in the SDT model of reasoning people are comparing distributions of arguments supporting different conclusions using a response criterion (see Fig. 1). The criterion determines the preference for Type 1 (rejecting a valid conclusion) or for Type 2 (accepting invalid conclusion) errors.

According to Dube et al. (2010), people use a simple heuristic of endorsing believable and rejecting unbelievable conclusions (response bias). However, application of this heuristic does not affect the accuracy of reasoning in any way. While the accuracy of reasoning remains constant, the type of error however changes after the application of different response criteria. This suggests that the previously reported beliefs-by-validity interaction is an artifact associated with the use of particular research methods and analysis. This perspective on reasoning resulted in protracted discussions in the field (Klauer and Kellen 2011; Singmann and Kellen 2014), and subsequent experiments showed that response bias was affected by the complexity of the task, cognitive abilities, and time pressure (Trippas et al. 2013). Summarizing this SDT approach, believability adds nothing to the validity of assessment, but is merely responsible for the general-affirmative or general-declining approach. The SDT approach entails that reasoning individuals use a response criterion to simplify the decision process by rejecting all unbelievable and accepting all believable conclusions. Simultaneously, they can process the validity of the conclusion and use its results to override the heuristic response, e.g., endorsing the unbelievable, but valid conclusion despite the general motivation to reject all unbelievable conclusions. Overriding might occur mostly for people with analytic cognitive style, as measured by the CRT (Trippas et al. 2015).

Indeed, the idea that the evaluation of believability and validity is independent and not interacting processes, can inspire revision of the traditional models of moral judgments. Compared to intuitive models of moral judgments, which suggest that individuals engage in reasoning to override the intuitive (presumably deontological) response, the new approach to moral judgment could predict that an individual selects a decision criterion to decide between two alternative actions. This criterion promotes a general-affirmation or general-declining approach to moral dilemmas and can be influenced, for example, by the severity or directness of harm, and characteristics of the people harmed or saved. Simultaneously, one can reflect on moral rules and on consequences, using this reflection to reconstruct the argument distribution and to override the response bias. Hence, one can chose the utilitarian response despite the motivation (induced by a conservative response criterion) to reject any directly harmful actions. By analogy, one can also choose the deontological response despite being motivated (induced by a liberal response criterion) to promote actions associated with utilitarian gain. Some initial research supports such a hypothesis. For example, it has been shown that increased severity of harm decreased the likelihood of making a utilitarian decision (Trémolière and De Neys 2013).

This recent approach to reasoning could thus be incorporated into theoretical models of moral judgments by reversing the roles of intuition and deliberation. Specifically, when reasoning one assesses the validity of a conclusion and the response criterion is affected by believability of a conclusion. By analogy, we propose that when solving a moral dilemma, one compares the utility of both alternative actions using the response criterion, and the criterion is affected by the affective response to the harm caused. The affective response is presumably rather a strong, “alarm-bell” reaction to causing harm (Greene et al. 2001). We cannot refute the idea that more subtle emotions which subsequently help to find more preferred alternative can also affect the response criterion. However, our main idea is that the criterion is predominantly affected by strong, vivid emotions.

Considering the above, we propose that the response criterion would affect moral judgments by promoting a general attitude toward acting or non-acting, but would not interact with the utility analysis. The presented SDT model of reasoning does not explain the characteristics of the validity (or utility), e.g., if is it a Type 1 or a Type 2 process. This issue can be discussed by the Logical Intuition theory, which we present in the following section.

### Conflict detection and intuitive estimation of consequences

In reasoning, believability and validity are sometimes in conflict. Both are claimed to have distinct cognitive mechanisms: Believability is assessed intuitively and validity reflectively (Evans et al. 1983; Newstead et al. 1992). The reflective source of the validity assessment has recently been questioned by several researchers. For a typical belief bias task (in which beliefs and validity are in conflict), a non-conflict version of the task (in which beliefs and validity cue the same response) is used as a benchmark. Simply put, despite neglecting validity in reasoning, individuals are less confident when validity conflicts with intuitive believability. De Neys and his colleagues (De Neys 2014; De Neys et al. 2010) have shown that individuals detect that they give biased responses (i.e., are endorsing believable but invalid conclusion), by declaring lower confidence and requiring more time to analyze the invalid conflict problem compared to an invalid no-conflict syllogism. This conflict detection is also observed when cognitive resources are limited by secondary task or when deciding with increased working memory load (De Neys and Schaeken 2007). Similar findings have been reported by Pennycook et al. (2015), in a study on the base rate neglect, where judgments of individuals are affected by social stereotypes when assessing probabilities (Tversky and Kahneman 1974). Pennycook and his colleagues (2014) have shown that despite people giving biased responses, individuals detect the conflict between stereotype and prior probabilities.

Logical Intuition, despite being available under limited cognitive resources, is claimed to have lower salience than belief-based intuition. Hence, individuals feel something is wrong with their intuitive response, but usually fail at making this doubt explicit and fail to override the intuitive response. Reflection, however, can increase the salience of the Logical Intuition and therefore enhance more logical reasoning (De Neys 2014; Handley and Trippas 2015). Thus, we suggest that processes which are typically claimed to be reflective (i.e., validity and probability assessment) are now suggested to be intuitive. Compared to the model of intuitive assessment of validity, an individual is expected to be able to assess (at least broadly) the consequences of considered moral alternatives (Dubljevic and Racine 2014). Our recent research suggests that deontological decision makers are also sensitive to consequences (Bialek and De Neys 2016b), including under cognitive load (Bialek and De Neys 2016a). Hence, individuals were as likely to intuitively represent consequences as to consider validity or probability assessments. Because consequences can be broadly estimated using Type 1 processes, it would thus not be required to deliberate in order to compare the utilities of alternatives, suggesting that utilitarian judgments could also have their origins in intuition.

### Response bias and moral judgments

The theories proposed by Haidt (2007) and Greene and Haidt (2002) suggest that deliberation can sometimes be implemented to solve a specific dilemma, which is consistent with the selective scrutiny model of belief bias in which individuals only reason when a conclusion is unbelievable. According to these theories, reflection promotes utilitarian judgments. However, as we have discussed, negative emotions associated with directly causing harm can induce individuals to adapt their response criterion along a conservative dimension, so it produces fewer false alarms, and subsequently cause individuals to demand greater utilitarian gain in order to make a utilitarian decision. Keeping the utilitarian gains constant across different dilemmas, while changing the required moral action, might increase the level of emotional response that is required for the sacrifices in particular dilemmas and increase the internal conflict and lower the metacognitive “feeling of rightness.” Low confidence might thus subsequently trigger reflection, which can either lead to trying to justify the intuitive response or to reconstruct the arguments so to find a more satisfying conclusion. In sum, we suggest that individuals engage in deliberation usually when the intuitive response does not provide enough confidence (“feeling of rightness”), regardless of whether this response is utilitarian or deontological. Most judgments however are made on the intuitive level, by broad argument representation, and its comparison using the response criterion.

There is still the remaining question regarding which response to rely on when the response criterion would be general affirmative or general declining. We created three model predictions which are different to those following from Greene’s model: (1) dilemma cued response, (2) moral inclinations cued response, and (3) promotion of the omission bias. Specifically, Greene’s model of moral judgments would suggest that the general-declining response criterion would promote deontological judgments, as these are assumed to be intuitive.

The first prediction is derived from the work of Kahane (Kahane 2012; Kahane et al. 2012), who described a problem, in which two of your friends are married, and you know that one of them had an affair. You believe that this will never happen again. The other member of the couple is suspicious and asks you whether you know anything about the unfaithful partner. Should you tell the truth, knowing it can destroy their marriage? In this dilemma it is suggested by Kahane that the utilitarian response is intuitive—regardless of what they ultimately choose, people intuitively focus on saving the marriage (utilitarian consideration) rather than on their duty to tell the truth (deontological consideration). Considering this example, we conclude that the response criterion would promote the decision which is cued by the particular dilemma.

The second prediction is derived from the work of Baron, who, after re-examining data from several studies on moral judgments, suggested that the first considered alternative depends from the person’s core moral preference (Baron et al. 2012). Supporting this claim he showed that the decisions that take the longest time in dilemmas are the ones suggested to be most difficult (the probability of deontological and utilitarian responding is about 50/50). Therefore, one can assume that more effort is required to override moral inclinations associated with a particular dilemma rather than overriding deontological intuitions with (utilitarian) reflection.

The third prediction is that general-declining response biases promote the omission bias (Spranca et al. 1991) presumably because individuals perceive omissions as non-decisions (Kordes-de Vaal 1996). This would result in greater preference for the default outcome, which in the majority of moral dilemmas is the deontological option.

In conclusion, recent findings suggest a need for a focused study on conflict detection in moral dilemmas. Our proposed approach to moral judgment is different to any dual-process model of moral judgment, whether sequential or parallel. We suggest that the judgment is based on competing intuitions according to a response criterion. Reflection can interfere and override the intuitive response, but does not necessarily lead to utilitarian judgments. Extending this topic, we have recently analyzed the impact of forced deliberation on moral judgments, showing that some type of reflection leads to deontological judgments and eliminates the impact of the type of harm caused (direct or indirect), while numerical reflection leads to utilitarian judgments and does not eliminate the impact of the type of harm (Bialek et al. 2016). This finding is contrary to the dual-process concept of moral judgments, but consistent with a SDT model of reasoning. Indeed, we suggest that the response bias can be a mechanism responsible for moral decision making and internal conflict resolution.
